# Diffuse Myocardial Interstitial Fibrosis and Dysfunction in Early Chronic Kidney Disease

**DOI:** 10.1016/j.amjcard.2017.11.041

**Published:** 2018-03-01

**Authors:** Manvir Kaur Hayer, Anna Marie Price, Boyang Liu, Shanat Baig, Charles Joseph Ferro, Jonathan Nicholas Townend, Richard Paul Steeds, Nicola Catherine Edwards

**Affiliations:** a Department of Nephrology, Queen Elizabeth Hospital Birmingham, Birmingham, United Kingdom; b Institute of Cardiovascular Sciences, School of Medicine and Dentistry, University of Birmingham, Birmingham, United Kingdom; c Department of Cardiology, Queen Elizabeth Hospital Birmingham, Birmingham, United Kingdom

## Abstract

Patients with chronic kidney disease (CKD) have a disproportionately high risk of cardiovascular (CV) morbidity and mortality from the very early stages of CKD. This excess risk is believed to be the result of myocardial disease commonly termed uremic cardiomyopathy (UC). It has been suggested that interstitial myocardial fibrosis progresses with advancing kidney disease and may be the key mediator of UC. This longitudinal study reports data on the myocardial structure and function of 30 patients with CKD with no known cardiovascular disease and healthy controls. All patients underwent cardiac magnetic resonance imaging including T1 mapping and late gadolinium enhancement (if estimated glomerular filtration rate > 30 ml/min/1.73 m^2^). Over a mean follow-up period of 2.7 ± 0.8 years, there was no change in left ventricular mass, volumes, ejection fraction, native myocardial T1 times, or extracellular volume with CKD or in healthy controls. Global longitudinal strain (20.6 ± 2.9 s^−1^ vs 19.8 ± 2.9 s^−1^, p = 0.03) and mitral annular planar systolic excursion (13 ± 2 mm vs 12 ± 2 mm, p = 0.009) decreased in CKD but were clinically insignificant. Midwall late gadolinium enhancement was present in 4 patients at baseline and was unchanged at follow-up. Renal function was stable in this cohort over follow-up (change in estimated glomerular filtration rate was −3 ml/min/1.73 m^2^) with no adverse clinical CV events. In conclusion, this study demonstrates that in a cohort of patients with stable CKD, left ventricular mass, native T1 times, and extracellular volume do not increase over a period of 2.7 years.

The increased cardiovascular morbidity and mortality of patients with chronic kidney disease (CKD) is due to a combination of myocardial disease, arteriosclerosis, and atherosclerosis.^1^, ^2^ The ability of cardiovascular magnetic resonance (CMR) imaging to characterize myocardial disease at the same time as providing accurate and reproducible assessment of left ventricular (LV) structure and function has redefined ‘renal cardiomyopathy.’ The subendocardial and midwall patterns of late gadolinium enhancement (LGE) in end-stage kidney disease were first reported in 2005.^3^ More recently, the emergence of T1 mapping techniques, without contrast, has demonstrated increased myocardial T1 relaxation times indicative of diffuse interstitial fibrosis in end-stage kidney disease.^4^, ^5^ Similar though less severe changes in T1 relaxation times and extracellular volume (ECV) expansion are also present in patients with early-stage CKD but not essential hypertension.^6^ It has been postulated that interstitial myocardial fibrosis is progressive with advancing CKD and that ‘uremic cardiomyopathy’ represents the end-stage phenotype. To date, however, there have been no longitudinal data to support this hypothesis. The aim of this study was to report 2- to 3-year follow-up CMR data from our cohort of patients with early-stage CKD and healthy controls to provide information on the natural history of these changes.

## Methods

Patients who participated in an observational study investigating the use of CMR T1 mapping in early-stage CKD during 2012 to 2014 were invited to participate in an ongoing study examining myocardial disease and LV fibrosis in CKD (NCT03176862).^6^ Inclusion criteria were as follows: CKD stage 2 (estimated glomerular filtration rate [eGFR] 60 to 89 ml/min/1.73 m^2^ with other evidence of kidney disease: proteinuria/hematuria/structural abnormality/genetic), stage 3 (eGFR 30 to 59 ml/min/1.73 m^2^), stage 4 (15 to 29 ml/min/1.73 m^2^), and stage 5 (<15 ml/min/1.73 m^2^, predialysis) with no history or symptoms of cardiovascular (CV) disease or diabetes. Healthy controls and hypertensive subjects in the original cohort were also invited to participate. The study was approved by the National Research Ethics Service—East Midlands (15/EM/0280) and all subjects gave written informed consent. Demographic, medical co-morbidities, and blood and proteinuria data were collected prospectively. Glomerular filtration rate was assessed using the CKD-EPI equation. CV events were defined as death from CV disease, myocardial infarction, stroke, peripheral vascular disease, or hospital admission with heart failure.

All subjects underwent CMR (1.5 T Siemens Avanto, Erlangen, Germany) with a phased array 18-channel body matrix coil and spine matrix coil. Imaging sequences for LV and right ventricular structure, function, and LV mass were acquired in line with standard protocols.^7^ Vertical long-axis and horizontal long-axis steady-state free precession cine images (retrospective electrocardiographic gating) were used to pilot the LV short-axis stack (SAX) acquired using serial cines (typical parameters were resolution of 40 to 50 milliseconds, repetition time of 3.2 milliseconds, echo time of 1.7 milliseconds, flip angle of 60°, field of view of 300 mm, in-plane resolution of 1.5 × 1.5 mm^2^, slice thickness of 7 mm with 3 mm gap, minimum 25 phases per cardiac cycle). T1 mapping was performed before and 15 minutes after gadolinium administration on the basal and mid-SAX slices in diastole using an electrocardiography-gated Modified Look-Locker Inversion recovery (MOLLI) sequence with a 3(3)5 sampling protocol (Siemens WIP 448). Typical acquisition parameters were pixel bandwidth of 977 Hz/pixel; echo time = 1.1 milliseconds; flip angle = 35°; matrix = 144 × 256; slice thickness of 6 mm. Motion correction and a nonlinear least-square curve fitting were performed with the set of images acquired at different inversion times to generate a parametric pixel-wise color T1 map to quantitatively measure the longitudinal myocardial relaxation time. LGE imaging was acquired in the corresponding SAX stack and LAX views using a standard inversion recovery sequence 7 to 10 minutes after 0.15 mmol/kg of gadolinium contrast bolus (Gadovist Bayer Health Care, Newbury, Berkshire, England). For the original study, ethical approval allowed administration of gadolinium to subjects with eGFR > 15 ml/min/1.73 m^2^. This dose was amended in the follow-up study to eGFR > 30 ml/min/1.73 m^2^ because of local policy change and guidance from the Medicines and Healthcare Products Regulatory Agency.

Offline analysis was performed by experienced operators (MK, NE) using CVi 42 software (version 5.3.4 cvi42, Circle Vascular Imaging, Calgary, Canada). Analysis of LV function, volume, and LV mass was performed with delineation of papillary muscles and trabeculations using thresholding. Tissue tracking in the 3 long-axis views was performed offline to assess 2-dimensional global longitudinal strain (GLS), strain rate, and early diastolic strain rate. Native and postcontrast myocardial T1 values were measured from parametric T1 maps. Endocardial and epicardial borders were manually drawn in the basal and mid short-axis slices with meticulous care to avoid partial voluming and blood pool contamination. Anterior and inferior septal borders were defined with semiautomated segmentalization of the LV in accordance with the AHA model.^8^ Average anteroseptal and inferoseptal T1 times were reported. ECV was calculated using validated formulas as previously described.^6^ Areas containing LGE, consistent with previously undiagnosed infarction or right ventricular insertion point LGE, were excluded from the T1 and ECV analysis.^9^

All T1 map studies were analyzed twice by a single blinded observer (MKH) for intraoperator reproducibility and a second blinded observer (AP) for interoperator reproducibility. Analysis was performed on studies anonymized to time and date by a third observer who assigned a unique number to all scans before analysis.

Continuous variables are expressed as mean ± SD if normally distributed or median (25th to 75th percentile) if non-normally distributed. Paired group comparisons for continuous data were made using the paired samples *t* test or the Wilcoxon signed-rank test for parametric and nonparametric data, respectively. Paired nominal data were compared using the McNemar test. Statistical tests were 2-tailed, and a p value <0.05 was considered to indicate statistical significance.

## Results

In total, 30 subjects with CKD from the original cohort (43 subjects) were followed up for a mean period of 2.7 ± 0.8 years. Baseline and follow-up demographic, biochemical, and treatment data for the patients with CKD are presented in Table 1. The mean age at baseline was 57 ± 10 years, and 60% of the participants studied were male. There was no difference between patients who returned for follow-up and those who did not (data not shown). The etiologies for CKD in the study cohort were as follows: primary glomerulopathy (37%), polycystic kidney disease (30%), vasculitis (13%), hypertension (7%), and others (13%). No patients experienced a CV event during follow-up. Using the National Institute for Health and Care Excellence (NICE) guidance, no patients had progressive CKD as defined by a sustained decrease in eGFR ≥ 25%/year or ≥15 ml/min/1.73 m^2^/year over the study period [median and interquartile range for change in eGFR −2 (−10.5 to 1.75)] but 7 patients did change their CKD category during the follow-up period.^10^ Eight healthy volunteers from the original cohort (43 subjects) agreed to be re-studied. There was no change in systolic blood pressure in the CKD or control groups, but diastolic blood pressure did increase in patients with CKD (Table 1).

**Table 1:** Demographic, biochemical, and treatment data

Variable	CKD Patients (n = 30)		p Value
	Baseline	Follow Up	
Body surface area (m^2^)	1.87 ± 0.17	1.89 ± 0.15	0.21
Body mass index (kg/m^2^)	27 ± 5	27 ± 3	0.66
Hemoglobin (g/L)	126 ± 17	133 ± 15	0.01
Hematocrit (%)	0.37 ± 0.05	0.39 ± 0.04	0.10
Serum creatinine (mg/dL)	1.71 ± 1.02	1.96 ± 1.75	0.03
N-terminal pro B Natriuretic peptide (ng/L)	81 (38–169)	85 (32–220)	0.29
Total cholesterol (mg/dL)	178 ± 39	193 ± 50	0.25
Estimated glomerular filtration rate (ml/min/1.73 m^2^)	53 ± 23	50 ± 23	0.02
CKD stage, *n*			0.03
1	2 (7%)	1 (3%)	
2	12 (40%)	11 (37%)	
3	10 (33%)	11 (37%)	
4	5 (17%)	4 (13%)	
5	1 (3%)	3 (10%)	
Urine albumin to creatinine ratio (mg/g)	51 (19–242)	70 (18–158)	0.72
Office systolic blood pressure (mmHg)	129 ± 12	132 ± 17	0.48
Office diastolic blood pressure (mmHg)	69 ± 11	77 ± 9	<0.01
Medications			
Angiotensin converting enzyme/angiotensin receptor blocker	25 (83%)	26 (87%)	0.25
Diuretic	7 (23%)	6 (20%)	0.38
Beta blocker	2 (7%)	3 (10%)	0.38
Calcium channel blocker	8 (27%)	9 (30%)	0.38
Statin	8 (27%)	12 (40%)	0.44

Data are frequency (%), mean ± SD, or median (interquartile range).

**Figure 1:**
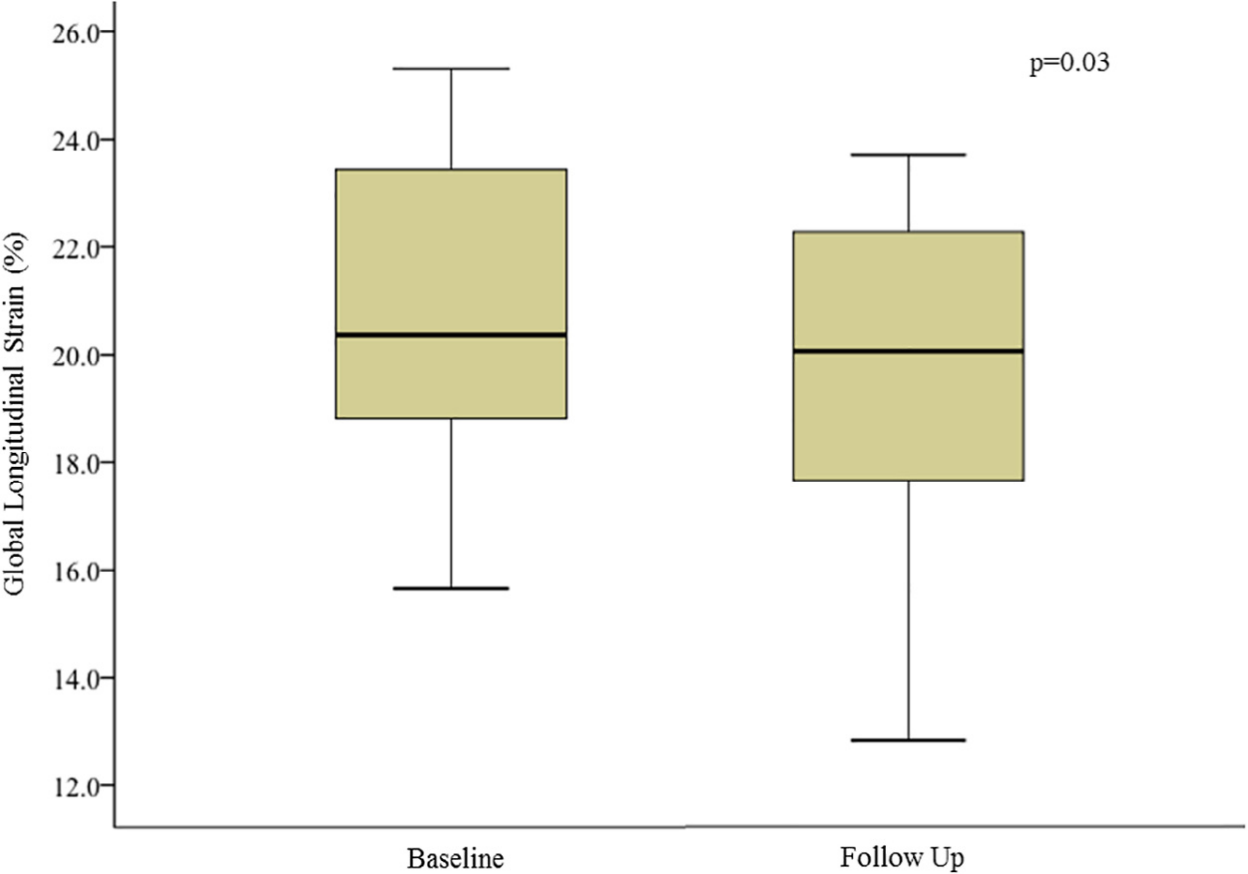
Change in global longitudinal strain between baseline and follow-up. The vertical line in each box represents the median, the boxes represent the 25th to 75th percentile, and the whiskers represent the minimum and maximum values in each dataset.

**Figure 2:**
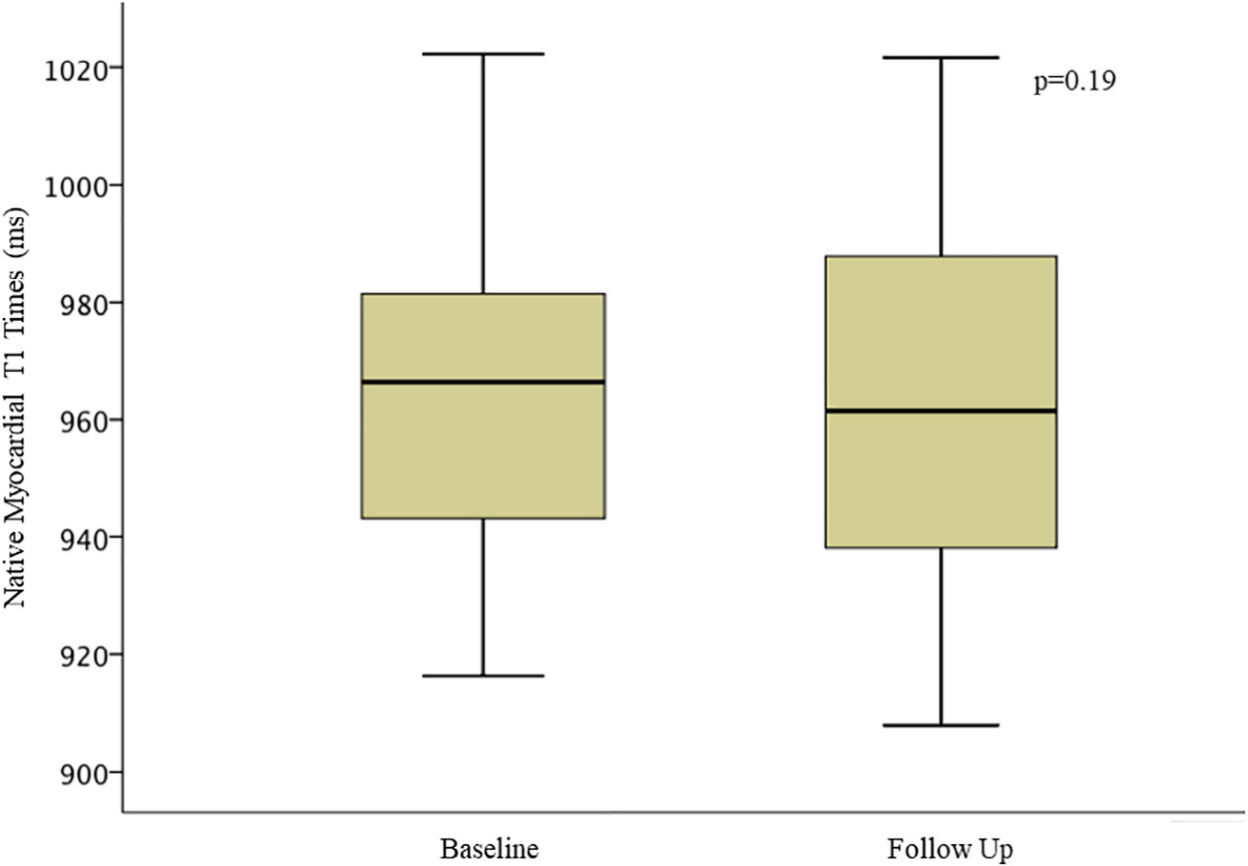
Change in native myocardial septal T1 times between baseline and follow-up. The vertical line in each box represents the median, the boxes represent the 25th to 75th percentile, and the whiskers represent the minimum and maximum values in each dataset.

Cardiac magnetic resonance imaging data for patients with CKD are presented in Table 2. There was no change in LV volumes, mass, or systolic function assessed using ejection fraction for the CKD or the control groups. A small reduction in mitral annular planar systolic excursion and GLS was observed, although values remained within the normal range (Figure 1). There was no change in myocardial T1 times (Figure 2) or ECV in either group. Because of changes in ethical approval, gadolinium was only administered to patients with eGFR > 30 ml/min/1.73 m^2^ (22 of 30 subjects) at follow-up. Midwall LGE was observed in 4 patients. None had subendocardial LGE. There were no new cases of myocardial LGE in either CKD or control groups over follow-up, but 4 patients with CKD did develop new focal right ventricular insertion point LGE (Table 2).

**Table 2:** Cardiovascular structure and function on cardiac magnetic resonance imaging

Variable	CKD Baseline	CKD Follow Up	p Value
Left ventricular ejection fraction (%)	69 ± 9	71 ± 6	0.14
Left ventricular end diastolic volume index (ml/m^2^)	67 ± 15	67 ± 11	0.92
Left ventricular end systolic volume index (ml/m^2^)	20 ± 8	22 ± 13	0.52
Left ventricular mass indexed (g/m^2^)	62 ± 14	62 ± 11	0.71
Native septal T1 time (ms)	967 ± 27	970 ± 26	0.19
Septal extracellular volume (%)*	26.1 ± 2.2	26.6 ± 1.7	0.33
Post contrast septal T1 time (ms)*	437 ± 37	448 ± 26	0.13
Presence of late gadolinium enhancement, n*			
Right ventricular insertion point	4 (13%)	8 (36%)	0.05
Mid wall/diffuse	4 (13%)	4 (18%)	-
Native T1 times in areas of right ventricular insertion point LGE (ms)	1043 ± 35	1063 ± 32	0.15
Native T1 time in areas of mid wall late gadolinium enhancement (ms)	1028 ± 40	1050 ± 51	0.55
Mitral annular planar systolic excursion (mm)	13 ± 2	12 ± 2	0.01
Global longitudinal strain (%)	20.6 ± 2.9	19.8 ± 2.9	0.03
Global longitudinal systolic strain rate (s^−1^)	1.12 ± 0.17	1.07 ± 0.22	0.26
Global longitudinal early diastolic strain rate (s^−1^)	0.92 ± 0.24	0.89 ± 0.26	0.57

Data are frequency (%) or mean ± SD. Values are indexed for body surface area.

Myocardial T1 times were assessed in the left ventricular septum from the basal and midventricular levels and averaged to yield the “septal T1 time.” Measurements excluded myocardial and right ventricular insertion point late gadolinium enhancement.

* Eight patients did not receive gadolinium contrast in the follow-up cardiovascular magnetic resonance study as eGFR had fallen to <30 ml/min/1.73 m^2^.

In subgroup analyses in the 4 patients with myocardial LGE at baseline, there was no increase in myocardial T1 times, or reduction in GLS and peak systolic strain rate at follow-up (data not shown). In those patients from the original study with native myocardial T1 values greater than the upper limit observed in controls (8 patients, T1 > 985 milliseconds), there was no change in myocardial T1, ECV, LV function, or LV mass. There was no systemic bias detected by Bland-Altman analysis between intra- and interoperator agreement for native myocardial T1 times. The mean intra- and interobserver differences [mean ± SD (95% limits of agreement)] were −1 ± 6 (−12 to 10) and −1 ± 7 (−13 to 11), respectively.

## Discussion

This study demonstrates that in a cohort of patients with predominantly stable CKD, with controlled blood pressure and without known CV disease or diabetes, myocardial T1 times and ECV do not change over 2.7 years. There is also stability of LV ejection fraction and LV mass. These data are encouraging in that they demonstrate stability in the CMR measurement of T1 relativity in the context of CKD. The data are also reassuring for patients with stable early CKD and provide further support for remote community follow-up of such patients provided that blood pressure and renal function are monitored.

This is the first longitudinal study evaluating T1 and ECV in early CKD. No progression of T1 times or ECV was found in subjects over 2.7 years. We cannot exclude progression of these parameters over longer time periods and we are not able to comment on the influence of declining renal function. In keeping with these findings, a recent small longitudinal study of 22 hemodialysis (HD) patients studied twice over 6 months did not show a change either in septal T1 times or in BNP.^11^ LV mass in this study fell with the introduction of HD, which may imply a greater regression of myocyte hypertrophy rather than change in the extracellular environment including fibrosis, as has been reported using ECV assessment after aortic valve replacement.^12^

The unexpected stability of CKD in the study prevents us from addressing our original hypothesis that deteriorating renal function promotes myocardial fibrosis, a key intermediary in the progression of UC. It should be noted that this is not a new concept, as the earliest reports of histology dating back to the late 1980s from postmortem and cardiac biopsies confirmed that extensive fibrosis is detected in HD patients.^13^ It is interesting that 3 groups who have published using T1 mapping in CKD have looked at different stages of disease (early stage 2 to 4, dialysis with short vintage 5.5 months and longer vintage 21 months). Although it is not possible to compare absolute T1 values directly because of different sequences and magnet strengths (native values being higher at 3 T), it does appear that any difference in T1 between controls and patients increases with severity of disease and dialysis vintage. Edwards et al reported higher native septal T1 times and ECV, with 14% showing evidence of irreversible fibrosis by LGE in patients in stage 2 to 4 CKD. The authors acknowledged a degree of overlap of T1 times with controls as has been reported with other disease entities.^6^ Rutherford et al showed higher septal and global T1 times, as well as a correlation with LV mass in HD patients with a short vintage compared with healthy volunteers but again an overlap of T1 times.^5^ However, data from a HD cohort with a long vintage showed native septal T1 times were all above the T1 times seen in healthy controls in support of progressive and more extensive fibrosis. Furthermore, 50% of HD patients had discrete midwall T1 signal comparable with that seen with LGE in DCM.^4^ Our data demonstrated myocardial LGE in only 4 subjects (13%) at baseline and follow-up but a significant increase in right ventricular insertion point LGE. The significance of this pattern of LGE is not fully understood, although it appears that the pattern has a lower risk profile than myocardial LGE in other disease cohorts.^14^ Autopsy examination has shown increased collagen and fat between fiber bundles (plexiform fibrosis), which is consistent with myocardial disarray but not pathologic fibrosis.^15^ It is clear that cross-sectional studies to date are, however, not definitive and these and our present study highlight the need for a large-scale, longitudinal follow-up study of patients with progressive CKD, for example, in those with high proteinuria, uncontrolled blood pressure, diabetes, and varying socioeconomic class.

GLS provides a potential functional biomarker for CKD cardiomyopathy. It provides a quantitative evaluation of ventricular global and regional function that has been linked to the extent of myocardial fibrosis and is a better predictor of mortality and adverse CV events in a range of conditions particularly when EF is within normal range.^16^, ^17^, ^18^, ^19^ We showed a very small reduction in GLS over time in the absence of a change in LV mass, systolic blood pressure, or BNP, but acknowledged that the values remain within the normal range and this finding is of questionable significance in this small cohort. Nevertheless, the statistically significant result seen with GLS raises the question whether strain may be a more sensitive imaging biomarker than native myocardial T1 times. Serum biomarkers of fibrosis also appear to be important in detecting and monitoring myocardial disease in CKD, with recent data confirming a trend with galectin-3 and GLS in HD patients.^5^

There are several limitations to acknowledge; the effects of progressive renal dysfunction on myocardial disease cannot be addressed from this study because of the stability of our patient's CKD. Our patients with mainly low levels of proteinuria, no diabetes, and well-controlled blood pressure were a relatively low-risk cohort and we note that other studies have reported considerable variability in rates of renal progression over such a time period.^20^ This is a small study and it is not possible to exclude selection bias in the 30 patients who returned for follow-up. However, the other 13 patients from the original study were all contacted and confirmed to be well and without reported progression to renal replacement therapy or CV events.

In conclusion, in patients with early-stage CKD, noninvasive imaging biomarkers of myocardial fibrosis do not progress if renal function remains stable. These findings are paralleled by stability of LV mass and systolic function.
